# Maternal Immunity and the Natural History of Congenital Human Cytomegalovirus Infection

**DOI:** 10.3390/v10080405

**Published:** 2018-08-03

**Authors:** William J. Britt

**Affiliations:** Departments of Pediatrics, Microbiology, and Neurobiology, University of Alabama School of Medicine, University of Alabama at Birmingham, Birmingham, AL 35294, USA; wbritt@peds.uab.edu

**Keywords:** human cytomegalovirus, congenital cytomegalovirus infection, maternal antiviral immunity, intrauterine infection

## Abstract

Congenital human cytomegalovirus (HCMV) is the most common viral infection of the developing fetus, and a significant cause of neurodevelopmental abnormalities in infants and children. Congenital HCMV infections account for an estimated 25% of all cases of hearing loss in the US. It has long been argued that maternal adaptive immune responses to HCMV can modify both the likelihood of intrauterine transmission of HCMV, and the severity of fetal infection and risk of long term sequelae in infected infants. Over the last two decades, multiple studies have challenged this paradigm, including findings that have demonstrated that the vast majority of infants with congenital HCMV infections in most populations are born to women with established immunity prior to conception. Furthermore, the incidence of clinically apparent congenital HCMV infection in infants born to immune and non-immune pregnant women appears to be similar. These findings from natural history studies have important implications for the design, development, and testing of prophylactic vaccines and biologics for this perinatal infection. This brief overview will provide a discussion of existing data from human natural history studies and animal models of congenital HCMV infections that have described the role of maternal immunity in the natural history of this perinatal infection.

## 1. Introduction

Congenital infection (present at birth) with human cytomegalovirus (HCMV; cCMV infection) is the most frequently reported viral infection in the newborn infant. The prevalence of this infection has been reported to range from 2/1000, to as high as 20/1000 live births [1,2,3,4,5]. Large studies in the US and in Brazil which employed rigorous screening programs have reported an overall prevalence of about 6/1000 live births [1,6]. However, the prevalence varies widely, depending on the characteristics of specific maternal population such as race, age, economic status, and co-existing sexually transmitted infections. This is illustrated by the very low prevalence of cCMV infections in northern Europe and in non-urban populations in the US, whereas the highest prevalence of this perinatal infection can be found in Africa, southern Asia, South America, and in some urban areas in the US [6]. Significant race-dependent disparities in the reported incidence of cCMV infections in the US suggest that additional undefined characteristics of maternal populations could contribute to the natural history of this perinatal infection [7]. A unique and as yet unexplained characteristic of cCMV infection is that its prevalence increases as the prevalence of HCMV infection increases in the maternal population, and fails to reach a level at which time the incidence of cCMV falls [8,9]. This is in direct contrast to congenital rubella syndrome in which once the rate of maternal seroimmunity to rubella reaches between 80–85%, the incidence of congenital rubella syndrome drops dramatically [10,11]. Similarly, the prevalence of congenital Zika syndrome in northeast Brazil dropped precipitously as the Zika virus seroprevalence rapidly increased to over 60% in this population [12]. Maternal infections during pregnancy rarely, if ever, result in a clinically identifiable infection, and exposures to HCMV occur continuously in pregnant women, in contrast to common viral respiratory pathogens that are associated with seasonal outbreaks. There are well-described exposure risks to HCMV that include exposure to young children, sexual activity, and living in crowded conditions [6,13,14,15,16,17,18,19]. The life-long persistence of HCMV in the infected host and its intermittent shedding in saliva, breast milk, and genital secretions provide an efficient mode of spread throughout populations. In contrast to the clinically asymptomatic infection in pregnant women, intrauterine transmission to the developing fetus can result in devastating consequences, including fetal loss. Fortunately, such severe infections are relatively uncommon, and about 90% of infants infected in-utero exhibit no findings in the newborn period that would allow their identification by physical examination. Yet, even infants without symptoms of cCMV infection are at risk for neurodevelopmental sequelae. Long term follow-up studies have determined that between 8–10% of infants with cCMV infection regardless of the presence or absence of symptoms at birth, will exhibit neurodevelopmental abnormalities [20,21]. The contribution of cCMV infections to disease in infants and children has been estimated to exceed that of the most common chromosomal disorder, trisomy 21, cystic fibrosis, and to be on the order of congenital heart disorders [22]. Hearing loss is the most common long term sequelae occurring in about 8–10% of infants and children with cCMV infections [23,24,25,26]. It is estimated that cCMV infections account for about 25–30% of all cases of hearing loss in children in the US [27]. Although the magnitude of contribution of cCMV infection to child health has been recognized for decades, including by the US Institute of Medicine, progress in the development of protective prophylactic vaccines and efficacious antiviral therapies has been limited. In the following sections, some of the more recent findings relative to the development of vaccines and biologics, to prevent or to reduce the incidence of damaging cCMV infections, will be reviewed in the context of decades old results. Together, observations from these studies illustrate the complexity of the interactions between the host and HCMV in this congenital infection, and suggest that newer approaches to understanding the relationship between HCMV and maternal adaptive immunity could be required for the development of effective prophylactic vaccines and biologics.

## 2. Epidemiology of cCMV Infections

**Figure 1:**
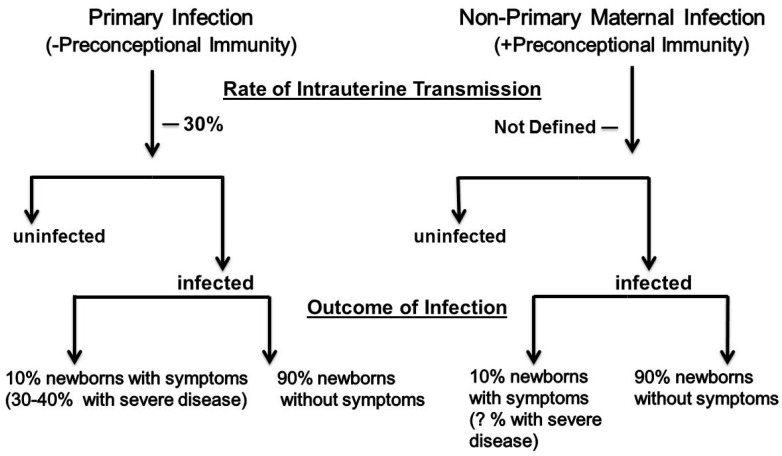
Congenital human cytomegalovirus (HCMV) infection during pregnancy: Classification of maternal infection and newborn outcome.

Over five decades of research have identified many of the parameters that define our current understanding of the natural history of cCMV infections. Key characteristics of this perinatal infection will be outlined to provide a framework for a discussion of the role of maternal immunity in this intrauterine infection. Maternal infections acquired during pregnancy in women without serological immunity to HCMV prior to conception have been designated as primary maternal infections (Figure 1). Infections in women with serological immunity prior to conception were initially described as recurrent infections, but following demonstration that women could be reinfected with new strains of HCMV during pregnancy and transmit those viruses to the developing fetus, this term has been replaced by a more accurate designation, non-primary maternal infection (Figure 1). The classification of maternal HCMV infection is informative because the type of maternal infection (primary vs. non-primary) has been used to stratify; (i) the risk of delivering an infant with cCMV infection (transmission to the developing fetus) and; (ii) the severity of the cCMV infection, including the presence of clinical abnormalities in the newborn period, and the risk for long term neurodevelopmental sequelae. It is in this context that the role of maternal immunity has been inferred from differences in the incidence of cCMV infection, and the severity of the infection in infants born to women with or without serological immunity who are infected during pregnancy. Unfortunately, results from many of the early studies that helped define paradigms in this perinatal infection were confounded by flaws in study design, diagnostics, and analytical laboratory methodologies with limited sensitivity, and cohorts that were not representative of the entire maternal population. As results from more contemporary studies have refined previous paradigms that defined that natural history of cCMV infections, several of the tenets of the epidemiology of cCMV infections have been challenged, including the role of maternal immunity in this perinatal infection.

The transmission rate of HCMV to the fetus following maternal primary infection during pregnancy has been reported to range between 20–70%, with the most studies reporting rates of around 30% (Figure 1) [4,21,28]. These rates vary dramatically, depending on the characteristics of maternal population, and have been shown to be increased in women with underlying deficits in adaptive immunity such as maternal populations with high rates of human immunodeficiency virus (HIV) infections [29,30,31]. Transmission rates appear to be highest after the mid-to-late second trimester of pregnancy, but the temporal relationship between maternal infection and the transmission event to the fetus is unknown in most cases [32]. Similarly, there appears to be a relationship between the severity of maternal infection, as measured by virus shedding in urine and blood and the risk of intrauterine transmission, although this relationship has not been precisely quantified in a sufficient number of women. Furthermore, some studies have not documented this relationship, perhaps reflecting the differences in study populations that have often included substantial numbers of patients that are prescreened and referred for suspected HCMV infection [33,34,35,36]. Importantly, maternal primary infections are rarely associated with any clinical findings, and results from many clinical studies were not prospective in their design, with infected women being identified retrospectively or through serological screening, resulting in significant risk for biased enrollment. In addition, commonly used serological assays often include arbitrary definitions of the duration of a maternal infection and thus cannot precisely define the timing of intrauterine transmission. Finally, prenatal screening for acquisition of HCMV during pregnancy is not widespread, and is limited to countries with liberal regulations governing pregnancy terminations. Even in the presence of these recognized limitations, well-designed prospective studies have reported the rates and risks for intrauterine transmission following primary maternal infection [4]. In contrast, the risks and the rate of intrauterine transmission following non-primary maternal infection remain essentially undefined. It has been argued for decades that the rate of transmission following non-primary infection is between 0.6–1%, based on the overall prevalence of cCMV in many maternal populations, with the underlying assumption being that all women with seroimmunity have an identical risk for a non-primary infection during pregnancy, and transmission to the fetus. There is no data to support this assumption, and exposure to HCMV varies considerably in different maternal populations, regardless of maternal immune status, as reflected by seroconversion rates in non-immune women that vary widely, ranging from 1–13%. Moreover, only about 20–30% of non-immune women who are infected during pregnancy, transmit the virus to their offspring, demonstrating significant variations in the risk of intrauterine transmission between individual women following primary infection during pregnancy. Thus, risk factors such as exposure to a new strain of virus capable of infecting seroimmune women resulting in intrauterine transmission, or potentially the chance of reactivation of a persistent infection and intrauterine transmission, remain undefined in women undergoing non-primary infection during pregnancy. As a result, claims that transmission rates in women with non-primary infections during pregnancy are lower than in women who acquire primary infections during pregnancy have not been supported by well designed, prospective studies. Recently, the rate of intrauterine transmission following non-primary maternal infection have been estimated, based on results from studies that have contained potentially biased enrollments secondary to participant selection and methodologies that are utilized to identify non-primary infections [34,37]. In one study, an analysis of a subset of women from single center maternal cohort was used to argue that the presence of immunity in women with non-primary infection decreased the risk of transmission by about four-fold, compared to women with primary infection; however, the subset of women used to define this effect of maternal immunity on transmission differed significantly in terms of the rates of primary and non-primary maternal infections, when compared to the remainder of women in this maternal cohort, suggesting that this finding could have resulted in an unrecognized bias in participants selected for the substudy [37]. A second study utilized the avidity of maternal IgG HCMV specific antibodies, and detection of virus shedding as measures of non-primary maternal infection to calculate a rate of transmission following non-primary maternal infection, in place of more conventional measures, such as preconceptional HCMV serological immunity to define non-primary maternal infection [34]. As a result, it is unclear whether these patients were classified accurately, particularly in view that investigators in Japan utilizing similar assays and a prospectively enrolled maternal cohort demonstrated that HCMV-specific IgG avidity assays cannot be used to accurately define maternal non-primary infection that leads to intrauterine transmission [38]. Thus, the quantitative impact of preconceptional immunity in intrauterine HCMV transmission remains undefined, and it will require carefully designed and implemented prospective studies that will also include carefully selected controls for known confounders that could impact the findings in cohorts of pregnant women.

In early studies, investigators classified cCMV infections based on the presence (symptomatic) or absence (asymptomatic) of clinical findings consistent with cCMV in the newborn period, a classification scheme that has been used to define both the severity of the intrauterine infection and risk of long-term sequelae in infected infants. Symptomatic cCMV infection is present in 5–10% of infants with cCMV infection, whereas over 90% of infants infected in utero exhibit no clinical symptoms or findings attributable to cCMV infections (Figure 1) [20,21]. Although early reports by Karin Alfhors and colleagues in Sweden described the frequent occurrence of clinically apparent cCMV infections following non-primary maternal HCMV infections, dogma from several natural history studies of cCMV infections argued that maternal immunity prior to pregnancy provided substantial protection from severe, symptomatic cCMV infections, and presumably long-term neurodevelopmental sequelae [39]. These studies resulted in the paradigm that symptomatic cCMV infections rarely, if ever, followed non-primary maternal infections. Since these original observations, multiple studies confirmed Alfhors’ findings and challenged this paradigm by documenting the occurrence of symptomatic cCMV infections in infants born to women with non-primary infections [2,24,38,40,41,42,43,44,45,46,47,48]. Similarly, the risk of long-term neurodevelopmental sequelae, particularly hearing loss, was shown in prospective studies to be comparable in infants born to women with primary HCMV, and women with non-primary HCMV infections during pregnancy (Table 1) [42,46]. Several reasons could account for the discrepancies between the findings from earlier studies, and those of more contemporary studies, including improvements in diagnostics, and therefore more accurate classification of the type of maternal infection; however, a review of some of the earlier studies suggests that both referral bias of study participants and the misclassification of the type of maternal infection likely contributed to the differences in results from these studies [20,49]. Some investigators have continued to argue that infants with the most severe manifestations of cCMV infection such as microcephaly, chorioretinitis, and major structural abnormalities in brain development can only result from primary maternal infections during pregnancy. However, there is limited definitive data to support this claim, as much of the data used to support this hypothesis is derived from clinical studies with significant design flaws, particularly selection biases, secondary to reliance on referral populations, as noted above. Moreover, accurate estimates of the incidence of severe manifestations in infants born following non-primary maternal infections derived from prospective studies with sufficient numbers of participants are not available, although numerous reports from a number of small series of patients have described that cCMV-infected infants with severe manifestations were born to mothers with non-primary infections. In addition, it should be noted that the incidence of severe cCMV infections is low, and infants with severe clinical findings, such as microcephaly and other evidence of structural brain damage, represent perhaps 3–5% of all cases of cCMV, or when expressed as prevalence, as 3–5/10,000 live births. Thus, most studies have not been powered with sufficient numbers of enrollees to definitively address this hypothesis.

## 3. Evidence That Adaptive Immunity Can Modify but not Prevent HCMV Infections

Clinical and laboratory findings that were initially reported from studies of allograft transplant recipients and subsequently in HIV-infected patients have provided convincing evidence of the importance of adaptive immunity and control of HCMV infections [50,51]. High mortality rates secondary to HCMV end organ damage have been consistently reported in these patient populations, particularly in the most immunocompromised populations such as those with HIV infectionand recipients of hematopoietic cell allografts [51,52,53,54,55,56,57,58,59,60,61]. In these patients, HCMV CD4+ and CD8+ T lymphocyte responses have been shown to be critical for the control of HCMV infection, and the reconstitution of these responses following successful anti-retroviral therapy, or in the post-transplant period, is associated with a decreased incidence of clinically apparent HCMV infections, as well as improved rates of overall mortality in these patients [51,62]. In addition, antiviral antibodies, including human monoclonal antibodies, have been shown to provide some clinical benefit in solid organ transplant recipients but there is limited data suggesting that antiviral antibodies are protective in the absence of T lymphocyte responses [63,64,65,66]. From these studies in patients with significant deficits in adaptive immune responses, the control of HCMV replication in immunocompetent individuals has been inferred to be secondary to adaptive immune responses, presumably HCMV-specific T lymphocyte responses and to a lesser extent, antiviral antibodies. To date, a quantifiable relationship between the level of HCMV-specific adaptive immune responses and the control of HCMV in normal immunocompetent hosts has not been defined, but it presumably extends over a broad range, as HCMV infection rarely results in clinical symptoms in children or adults, and secondly, when quantified, there is a broad range of T lymphocyte and antiviral antibody responses to HCMV in populations of normal individuals. Furthermore, in most studies these values were derived without precise information of the viral load in relevant compartments. Thus, it has been difficult to establish a level of adaptive immunity to HCMV that must be achieved by prophylactic vaccines, or passively administered biologics to be considered as protective. Finally, this discussion has not included a description of the importance of innate immune responses to HCMV, particularly NK cells. This arm of the immune response to HCMV is critical for the effective control of HCMV infection, as illustrated by the susceptibility of patients with deficits in NK responses to severe HCMV infections [67].

Animal models of HCMV infection including rodents, guinea pigs, and non-human primates have provided data consistent with the importance of adaptive immunity and the control of HCMV [68,69,70,71,72,73,74,75,76,77,78]. These models have utilized a variety of approaches to define the relative contribution of HCMV-specific T lymphocyte responses and antiviral antibodies in the control of species-specific CMV infections under the conditions of immune suppression that mimic those following transplantation in humans and lentivirus infections that model HIV/AIDS [78,79]. Depending on the experimental animal model, either arm of the adaptive immune system has been shown to provide some level of protection from uncontrolled virus replication and disease, suggesting that the redundancy in protective adaptive immune responses to HCMV could be required for the optimal control of this infection. However, it is important to note that attempts to induce or to provide sterilizing immunity in animal models have generally been unsuccessful. In fact, the capacity to readily re-infect previously infected rhesus macaques has been exploited to develop rhesus CMV (RhCMV) as a vector to deliver vaccines for both simian immunodeficiency virusand M. tuberculosis [80,81]. Although each of these model systems suffer some limitations and fail to recapitulate all facets of human infection with HCMV, findings in these systems have provided significant and often unexpected insight into mechanisms of protective responses to HCMV that almost certainly would not have been identified in human studies. 

Informative animal models of cCMV infections have been developed in guinea pigs and non-human primates, secondary to shared structural characteristics of hemochorial placentas that are present in all three species. Although a similar placental structure is present in rodents, transplacental transmission in rodents following peripheral inoculation has only been described in severely immunocompromised animals [82]. Furthermore, intrauterine transmission of RhCMV has only been recently described in severely immunocompromised pregnant rhesus macaques [83]. Thus, the bulk of findings describing the role of adaptive immunity in both intrauterine transmission and disease have been derived from studies in guinea pigs. In this model, the role of both antiviral antibodies and prophylactic vaccine-inducing immunity in protection from transmission, and damage to the developing embryo have been reported. Initial studies using immune and non-immune pregnant guinea pigs have demonstrated that immunity that was established after natural infection was protective in this model [84,85]. Using this model, Harrison demonstrated that adjuvanted affinity purified guinea pig CMV (gpCMV) glycoprotein B could limit the severity of infection in pregnant guinea pigs and improve pregnancy outcomes, including reducing the rate of congenital infection [86]. Subsequently, multiple studies have refined these initial findings and have provided evidence of protective immunity induced by: (i) gpCMV envelope glycoproteins, or combined with gpCMV tegument proteins, (ii) vectored recombinant envelope glycoproteins and tegument proteins, and (iii) replication-defective recombinant gpCMV [87,88,89,90,91]. In almost all cases, gpCMV vaccines provided some level of protection from severe maternal infection, pregnancy loss, and the runting of the offspring. In addition, in several studies, significant protection was provided by the passive transfer of polyvalent anti-gpCMV serum, anti-gpCMV gB antisera, and by monoclonal antibodies directed at gpCMV gH/gL [92,93,94]. One characteristic of the guinea pig model that differs significantly from HCMV infection in pregnant women is that in order to achieve reproducible transplacental transmission of gpCMV, pregnant guinea pigs must be inoculated with sufficient amounts of virus to induce significant symptomatic infections, often with appreciable maternal mortality and embryo loss. Furthermore, there is often considerable variability in the prevention of congenital gpCMV infection of pups from infected mothers, as compared to more consistent effects in the reduction of embryo loss and runting of newborn pups. These findings raise the possibility that in many of these studies, the major impact of individual vaccine preparations could be explained by modifications of the severity of gpCMV infection in the pregnant dams, and potentially the function of the placenta. In fact, investigators in Japan have provided evidence that the protective activity of an adenovirus gpCMVgB vaccine was most consistent with modification of placental infections with gpCMV [95]. Even with the limitations of this model, overall, the studies in guinea pigs have provided a considerable body of literature that suggests that in a controlled experimental setting, gpCMV induced immunity can modify maternal gpCMV infection, and improves outcomes of pregnancy.

As noted above, attempts to establish a model of human cCMV infection in immunocompetent non-human primates has not been reproducibly successful until recently, when investigators utilized CD4+ T lymphocyte depleted and severely immunocompromised rhesus macaques as a model system for cCMV infection [83]. In this model, RhCMV infection is induced in the CD4+ T lymphocyte-depleted pregnant macaques. Similar to observations in the guinea pig model, infection in the CD4+ T lymphocyte-depleted dams is severe and results in a reported maternal mortality rate of about 50% and 75% fetal loss [83]. However, in the surviving pregnant macaques and in seronegative immunocompetent pregnant macaques, intrauterine transmission of RhCMV as defined by the detection of Rhesus CMV DNA by nucleic acid amplification of amniotic fluid, but not recovery of infectious virus, could be demonstrated [83]. Endogenous immunity cannot be easily studied in this system, but in a study with a limited number of animals, passive transfer of RhCMV hyperimmune antiserum but not immune serum, was suggested to provide protection from severe maternal infection, placental damage, and transmission to the developing fetal macaque [83]. Together with results from studies in guinea pigs, these findings have argued that antiviral antibodies alone could be protective in cCMV infection. Although the findings from studies in this novel non-human primate model of cCMV infection argued in support of a role of protective antiviral antibody responses in limiting intrauterine transmission, the use of severely immune deficient animals and the passive transfer of antiviral antibodies nearly co-incident with virus infection, raises several questions about the relevance of this model to human cCMV infection, in which almost all women are not immunocompromised and the vast majority of infants with cCMV are born to women with existing preconceptional seroimmunity. Furthermore, the findings in non-human primates have been in contrast to the reported failure of hyperimmune globulin to prevent intrauterine transmission of HCMV in pregnant women undergoing primary HCMV infection [96,97]. However, it should also be noted that results from a recent study in a small group of pregnant women with primary HCMV infection argued that repeated administration of immune globulin can prevent intrauterine transmission [98]. When viewed with existing data, it could be argued that results, similar to those described in non-human primates, have also been described in guinea pigs, therefore providing an alternative animal model system to the more costly non-human primate model. In summary, animal model systems have provided new insights into the potential role(s) of adaptive immunity in two important aspects of cCMV, intrauterine transmission and the severity of intrauterine infection, as well as potentially translatable strategies for induction of protective immune responses. However, a definitive understanding of the role of adaptive immune responses and cCMV in humans will likely not be revealed by studies in current animal models of cCMV infections, but instead will require more directed studies using validated specimens, and epidemiological data from well-characterized maternal cohorts derived from carefully designed prospective studies.

## 4. Adaptive Antiviral Responses and Intrauterine Transmission of HCMV

Much of the existing data on the role of antiviral adaptive immune responses in the modification of the risk or severity of cCMV infections has been generated from studies of antiviral antibody responses, presumably secondary to technical issues surrounding the preservation of functional peripheral blood monocytes from a sufficient numbers of women enrolled in clinical studies. Findings from women with symptomatic HCMV infections have provided significant insight into potential correlates of protective adaptive immunity in cCMV infections; however, symptomatic HCMV infection occurs only in a small minority of pregnant women; thus the generalizability of these findings remains uncertain. Furthermore, the vast majority of cCMV infections occur in infants born to women with preconceptional adaptive immunity to HCMV, suggesting that findings from the analysis of the immune responses in pregnant women undergoing primary infection may be limited to this type of maternal HCMV infection. With this understanding of the limitations in our current understanding of the role of antiviral antibodies in modulating intrauterine transmission of HCMV, several findings have suggested potential characteristics of protective responses. These include decades-old as well as more contemporary studies that have demonstrated antiviral antibody responses to large numbers of virus-encoded structural and non-structural proteins during primary and non-primary HCMV infection in women of childbearing age [99]. Not surprisingly, these studies have failed to consistently define qualitative differences in these responses that could account for protection from intrauterine transmission, with the exception of studies in a small number of pregnant women that have argued that early antibody responses to the gH containing pentamer (gH/gL/UL128-131) complex correlated with protection from intrauterine transmission [100]. In other studies composed of similarly small numbers of prospectively enrolled patients, and in studies of women with symptomatic infection, intrauterine transmission in pregnant women with primary infection has been associated with increased levels of antiviral antibodies, and in some cases, higher responses have been correlated with higher viral loads [99,100]. Quantitation of antiviral responses in a group of women with primary infection during pregnancy demonstrated that infected women who did not transmit virus developed higher avidity antibodies, including those to gB, earlier than women with primary infection who transmitted virus to their offspring [100,101,102]. This increased avidity was specifically associated with increased levels of virus-neutralizing antibodies [101]. In other studies in women with primary infection, some with symptomatic infection, the presence of high avidity antiviral antibodies at early times in pregnancy was associated with decreased rates of intrauterine transmission [103]. As noted above, the potential role of anti-pentamer antibodies, and their capacity to limit virus entry into endothelial and epithelial cells, including cell-to-cell spread, has been argued to have a critical role in prevention of intrauterine transmission [100]. Importantly, these investigators have demonstrated differences in the quality and kinetics of responses to the pentamer complex between women who transmit and those who do not transmit HCMV to their fetuses [100]. Together with observations demonstrating differences in the kinetics of the development of high avidity, antiviral antibodies in women following primary infection has suggested that individual differences in the development of antiviral responses to HCMV infection could contribute to the variability in intrauterine transmission that has been observed, in studies of pregnant women. Because the variability between individuals is significant, it is unlikely that the use of serological assays to quantify the kinetics of high avidity antibody development will be an informative method to identify individual women with increased risk for intrauterine transmission [102]. Lastly, it is important to view these findings in women with primary infections in the context of findings in women with preconceptional immunity who deliver infants with cCMV infections, as recent studies have failed to reveal significant differences in virus-neutralizing antibody responses between women who transmit HCMV to their offspring, and control women from the same maternal population who do not transmit HCMV [104]. Thus, quantifiable differences in the kinetics and specificities of antiviral antibodies, as measured by current serological methodologies, are unlikely to explain differences in intrauterine transmission of HCMV in pregnant women.

In a more limited number of studies of T lymphocyte responses to HCMV following primary maternal infection in pregnancy, there appears to be a delay in the kinetics of development of HCMV-specific CD4+ T lymphocyte responses in women who transmit virus to their fetuses, as compared to women who do not transmit, and in one study, quantitative differences were observed between the number of pp65-specific ELISPOT-positive T cells in women who transmitted virus, as compared to non-transmitting mothers [105,106]. More recently, this same group of investigators utilizing a different assay system reported no significant differences in CD4+ or CD8+ T lymphocyte reactivity for three different virus-encoded proteins (IE-1, pp65, gH/gL/pUL128; gB) and a lysate of CMV-infected cells between women with symptomatic primary infection who transmitted viruses to their offspring, and women who did not transmit the virus [35]. In this study, the authors did suggest that there was a decreased frequency of CD4+ T lymphocytes with a long-term memory phenotype (IL-7R+) in women who transmitted virus to their offspring, suggesting that rapid establishment of CD4+ T lymphocytes of this phenotype could provide some protection from intrauterine transmission [35]. These findings also differed somewhat from other studies that argued that quantitative differences in HCMV specific T lymphocyte responses were associated with intrauterine transmission in women undergoing primary infection during pregnancy [106,107]. While these studies have provided conflicting data on the importance of early HCMV specific T lymphocyte responses as a correlate of protection from intrauterine transmission, studies of virus specific T lymphocyte responses in pregnant women have provided more robust correlations between HCMV specific CD4+ T lymphocyte responses and intrauterine transmission, than HCMV-specific CD8+ T lymphocyte responses. These findings contrast with the extensive literature on the role of CD8+ T lymphocyte responses in the outcome of allograft recipients infected with HCMV. 

## 5. Adaptive Antiviral Responses and the Severity and Long Term Outcome of cCMV Infection

Early studies of the natural history of cCMV infections quickly identified the prognostic importance of severe, symptomatic infections in newborns with cCMV. These studies detailed a spectrum of clinical findings associated with end-organ disease in these infants, including hepatitis, splenomegaly, decreased platelet counts, and a number of findings of central nervous system damage. Subsequent studies have also included significant intrauterine growth retardation as a finding in infants with symptomatic cCMV infections. In many of the early studies, infants with symptomatic infections were often referred from non-study populations and when included in study populations, likely biased the findings from these studies [20]. As a result, the paradigm that severe, symptomatic cCMV infections followed only primary maternal infections became established, even though as noted previously, investigators in Sweden reported that severe, symptomatic cCMV infections with adverse long term outcomes could follow non-primary maternal infections [39]. From these data, it was inferred that maternal immunity could modify intrauterine infection and prevent severe cCMV infection and end organ disease [108]. Subsequently, carefully designed and implemented prospective studies demonstrated that symptomatic cCMV infections could follow non-primary maternal infections and perhaps more importantly, that long term neurological sequelae could develop in infants infected following non-primary maternal infections (Table 1) [2,38,41,42]. As a result, the dogma that the presence of preconceptional maternal immunity can protect from damaging fetal HCMV infection has been challenged. Yet it is difficult to dismiss the possibility that effective control of HCMV infection in pregnant women can limit either the amount or the virulence of viral populations that infect the placenta, and subsequently infect the fetus. Furthermore, potent antiviral antibodies transferred to the developing fetus could limit dissemination and potentially end organ damage, an observation that was first reported in a study of transfusion-acquired HCMV in premature infants [109]. In addition, antiviral antibodies have been shown to be protective in animal models of cCMV infection [93,110,111,112]. Thus, maternal immunity likely does impact the outcome of fetal infection with HCMV, but quantifying this effect has been difficult in human studies. Although a recent study has demonstrated that repeated doses of hyperimmune globulin can prevent of intrauterine transmission during primary maternal infection, the potential of non-antiviral and unrecognized off-target effects that could follow infusion of large amounts of polyvalent IgGs confounds the interpretation of findings from this study [98]. Direct evidence of protective antiviral antibody activity in humans will likely require an effective and well-defined biologic, such as a monoclonal antibody that could prevent virus dissemination and end organ disease. Attempts to study this question in a prospective clinical observational study without enrollment biases, such as referred patients or patients identified by screening, will require large numbers of enrollees to detect differences in rare events such as delivery of an infant with clinical symptoms of cCMV infection that may occur in 1/2000–3000 live births, and even larger numbers to detect the modification but not the prevention of symptomatic infections. Finally, there is little published data to suggest that differences in the magnitude or characteristics of maternal HCMV T lymphocyte responses contribute to either the short-term or long-term outcomes of intrauterine HCMV infection. 

## 6. The Impact of Adaptive Immunity on cCMV Infections: Lessons from Vaccine Trials

As has been noted in the previous sections, understanding and quantifying the role of adaptive immune responses in human specimens from observational trials is filled with confounding variables that are often unforeseen. A much more definitive approach would be to establish protective responses by a prophylactic vaccine, followed by quantitation and characterization of those responses. This approach would also potentially allow identification of protective immune responses that are not currently measured in conventional assays of adaptive immune responses. Several early clinical trials of vaccines to provide protective adaptive immunity by prophylactic vaccines have been reported. These include a replication competent but attenuated virus, and an adjuvanted subunit vaccine consisting of a recombinant-derived gB [113,114]. The recombinant gB vaccine has been the most well-studied candidate vaccine. In a study of seronegative women, immunization with this preparation induced both antibody and T lymphocyte responses to gB [114,115]. Results for the clinical trial reported approximately 50% protection from infection in this population, and although there was statistical significance between infection in the vaccine and non-vaccine group, many investigators view the reported difference as being transient and less than statistically robust [114]. A follow-up study utilizing the same vaccine preparation in non-immune adolescent females failed to demonstrate a statistical difference between controls and vaccine recipients in terms of infection [116]. Although development of this vaccine has not progressed beyond these studies, serum specimens from women enrolled in the first study have been analyzed for correlates of protection [117]. There appears to be little difference between women whom received the vaccine and seropositive control patients in terms of the quantity of antibodies that are reactive with gB, that were produced following immunization; however, there was limited neutralizing capacity against unrelated strains of HCMV, as well as decreased binding activity for regions of gB that were shown to be targets of virus neutralizing antibodies in serum from the vaccine recipients, as compared to the controls [117]. Interestingly, and in contrast to previous studies in women infected with HCMV during pregnancy, the gB vaccine induced higher titers of IgG3 antibodies that were reactive with the cytosolic antigenic domain of gB, AD-3, than those observed in individuals with natural infection [117,118,119]. Lastly, the authors of this study speculated that non-neutralizing antibody functions induced by vaccine could impart some of the protective activity that had been ascribed to this vaccine preparation. Although findings and conclusions provided by this study are provocative, the minimal level of protection induced by this vaccine and the pitfalls that can influence the outcome of vaccine trials in pregnant women, including the impact of unintended counseling to limit exposure to HCMV, would argue that additional studies will be required before any definition of protective responses can be gleaned from the analysis of vaccine-induced antibodies from participants in this HCMV vaccine trial.

## 7. Conclusions

Numerous examples in both experimental animal models and in human populations have provided evidence for a significant role of adaptive immune responses and the control of HCMV replication and dissemination. Correlates between immune responses and outcomes have been identified for both antiviral antibodies and HCMV T lymphocyte responses in immunocompromised patients that also are consistent with findings from studies in experimental animal models. In contrast, such correlates have been difficult to unequivocally demonstrate in pregnant women infected with HCMV during pregnancy. Much effort has been placed on understanding the role of antiviral antibodies in prevention of maternal to fetal transmission and severe intrauterine infection. To date, there is little convincing data of qualitative or quantitative antiviral antibody responses that can be consistently correlated with protection from transmission or fetal disease. Several potential explanations have been suggested, including the inability to measure functionally important antiviral antibody responses with conventional assays that have been employed in most studies, an undefined role of the placenta in the activity of antiviral antibodies (and potentially HCMV specific T lymphocytes) in the prevention of maternal to fetal transmission and severe intrauterine infections, the potential importance of viral genetic diversity in limiting effective antiviral function, and finally, the contribution of viral immune evasion functions in blunting adaptive immune control. Finally, even though animal models have provided an important insight into potential immune correlates of protective responses, the heterogeneity of human populations and the fundamental differences between repeated community exposures to potentially swarm-like populations of HCMV, as compared to genetically homogenous viruses utilized as a single challenge in animal models, raise questions about relevance of findings from animal models. Thus, it could be argued that only the analysis of specimens from maternal cohorts enrolled in well-designed studies will provide definitive data on the nature of protective adaptive responses that modify the natural history of cCMV infections.

## Figures and Tables

**Table 1:** Maternal immune status and long-term outcome of cCMV infection.

Permanent Sequelae ^1^	Primary Maternal Infection	Non-Primary Maternal Infection
Neurodevelopmental (non-hearing)	9% (8/90)	39% (15/38)
Hearing Loss	11% (20/187)	14% (21/153)

^1^ Primary data provided in references [24,42,44,46,48].
